# Redox and Metabolic Regulation of Intestinal Barrier Function and Associated Disorders

**DOI:** 10.3390/ijms232214463

**Published:** 2022-11-21

**Authors:** Pei-Yun Lin, Arnold Stern, Hsin-Hsin Peng, Jiun-Han Chen, Hung-Chi Yang

**Affiliations:** 1 Department of Laboratory, Taipei City Hospital Yang Ming Branch, Taipei 11146, Taiwan; 2 Grossman School of Medicine, New York University, New York, NY 10016, USA; 3 Center for Molecular and Clinical Immunology, Chang Gung University, Taoyuan 33302, Taiwan; 4 Division of Chinese Medicine Obstetrics and Gynecology, Department of Traditional Chinese Medicine, Chang Gung Memorial Hospital at Linkou, Taoyuan 33305, Taiwan; 5 Department of Medical Laboratory Science and Biotechnology, Yuanpei University of Medical Technology, Hsinchu 30015, Taiwan

**Keywords:** tight junction, intestinal barrier, leaky gut syndrome, reactive oxygen species, pentose phosphate pathway

## Abstract

The intestinal epithelium forms a physical barrier assembled by intercellular junctions, preventing luminal pathogens and toxins from crossing it. The integrity of tight junctions is critical for maintaining intestinal health as the breakdown of tight junction proteins leads to various disorders. Redox reactions are closely associated with energy metabolism. Understanding the regulation of tight junctions by cellular metabolism and redox status in cells may lead to the identification of potential targets for therapeutic interventions. In vitro and in vivo models have been utilized in investigating intestinal barrier dysfunction and in particular the free-living soil nematode, *Caenorhabditis elegans*, may be an important alternative to mammalian models because of its convenience of culture, transparent body for microscopy, short generation time, invariant cell lineage and tractable genetics.

## 1. Introduction

### 1.1. How the Tight Junctions “Break Down”, Resulting in Diseases

The healthy and balanced state of the intestine, known as intestinal homeostasis, is determined by gut microbiota, an intact intestinal epithelium and host immunity. In particular, maintenance of intestinal homeostasis depends on the integrity of the intestinal epithelium, which is supported by junctional proteins forming a physical barrier and connecting adjacent epithelial cells. Compromised barrier function leads to several pathologic conditions, including the leaky gut syndrome, neurodegeneration, inflammatory bowel disease (IBD), celiac disease, irritable bowel syndrome, obesity, diabetes and colorectal cancer [1].

Leaky gut syndrome is also known as the intestinal wall leakage syndrome. The healthy intestinal mucosa is a fine and tightly meshed structure. Upon damage and inflammation, the meshed structure loosens, resulting in a breakdown of barrier integrity [2]. The weakened tight junctions (TJ) allows for leaking from the intestinal mucosa into the blood and lymphatic circulation of allergens, microorganisms and incompletely digested macromolecular metabolites (such as gluten, proteins, peptides) and toxins (such as heavy metals or pesticides), all of which causes inflammation [3]. A leaky gut has a comprehensive impact on human health. The causes of leakage are ascribed to microbial infection, chronic allergen exposure, oxidative stress and dysbiosis [4,5,6,7] (Table 1).

### 1.2. Redox Systems That Affect TJ

Reactive species (RS), including reactive oxygen species (ROS) and reactive nitrogen species (RNS), are produced by the major NADPH-dependent enzymes, such as the NADPH oxidases (NOX) and the nitric oxide synthases (NOS). RS mediate inflammatory responses and can be generated in large quantity by inflammatory cells and macrophages. Intracellular RS affect intestinal epithelial function through modulating TJ [4,16]. The RS-producing enzymes modulate the structure and function of target proteins by modifying cysteine residues [17]. Alteration, either excess or deficiency, of RS disrupts the redox balance leading to the development of gut disorders, including intestinal cell dysfunction [4], autoimmune diseases [18], colitis [19] and IBD [20]. Likewise, low-molecular weight signaling molecules with reactive and diffusible properties influence intestinal health. Hydrogen sulfide (H_2_S) produced by intestinal microbiota and/or colonocytes affects the physiology of the host and influences the pathophysiology of gut-associated disorders [21]. While a minimum amount of H_2_S has anti-inflammatory effects [22], higher levels of luminal H_2_S are detrimental to mucus layer integrity and are involved in colorectal carcinogenesis [23]. Carbon monoxide (CO), a product of heme oxygenase, is associated with anti-inflammatory, anti-apoptotic and cytoprotective effects [24]. CO protects intestinal epithelial integrity by up-regulating TJ protein expression and reducing pro-inflammatory cytokines. The actions of CO may have therapeutic usefulness in sepsis and ulcerative colitis [25].

### 1.3. Permeability Barrier Related to Tight Junction Structure

The human intestine is a unique organ composed of the intestinal epithelium, microbiota, and an immune system. The intestinal epithelium, a part of the intestinal mucosa, is composed of a single layer of enterocytes which is supported by the lamina propria and the muscularis mucosae. A monolayer of epithelial cells forms a physical barrier joined by intercellular junctions. The three apical junctions are the TJ, the adherens junctions and the desmosomes. While TJ allows the passage of ions, water and solutes, this relatively impermeable membrane prevents luminal microorganisms, antigens and xenobiotics from reaching the serosa and entering the blood circulation. The function of the TJ is of importance in intestinal health, as a defective intestinal barrier leads to diseases, including bacterial enteritis and IBD [1].

**Figure 1:**
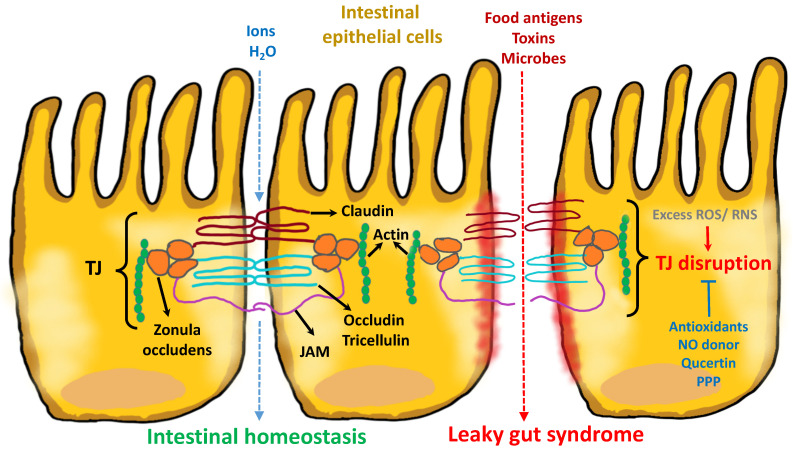
Schematic diagram showing the assembly of tight junctions (TJ) and the factors affecting TJ integrity. Intestinal homeostasis is maintained by an intact TJ, which is organized by occludin, claudin, JAM, and Tricellulin as well as zonula occludens in the intestinal epithelium. Redox imbalance due to toxic reactive species (ROS and RNS) disrupts the barrier function of the TJ, leading to enhanced permeability to incompletely digested food antigens, xenobiotics, and microbial products. Compromised barrier integrity eventually causes chronic inflammation in the intestine. In contrast, factors with antioxidant properties can ameliorate TJ damage and are potential therapeutic targets.

The function of the TJ is determined by the expression level, distribution and phosphorylation of the TJ proteins. TJ are formed by the assembly of different integral transmembrane proteins that occupy the paracellular zone and control the permeability of paracellular transport. Intestinal cells have four primary groups of transmembrane proteins, including occludin, claudins, junctional adhesion molecules (JAM) and tricellulin [26]. Structurally, occludin and claudins contain four transmembrane domains with the N-terminus and the C-terminus in the cytoplasm. JAM has only one transmembrane domain. These extracellular loops interact with the same transmembrane proteins of adjacent cells (Figure 1).

Occludin, abundant at cell–cell contact points, is required for the organization and maintenance of the TJ. Occludin is phosphorylated during oxidative stress-induced TJ disruption [27]. Phosphorylated occludin localizes in the membrane, while minimal phosphorylation of occludin is found in the cytoplasm. In vitro study shows that phosphorylation of occludin by *c-Src* attenuates the interaction with ZO-1 and destabilizes the assembly of the TJ [10]. Ubiquitination and phosphorylation of occludin are required for TJ trafficking and permeability in endothelial cells, indicating a regulatory role of occludin in TJ [28]. Despite the normal structure and function of the TJ in occludin-knockout mice, they display chronic inflammation, hyperplasia of gastric epithelial cells and multiple growth defects [29]. A single knockout of occludin or tricellulin has minimal impact on the morphology and permeability of the TJ, whereas double knockout of these proteins reduces cross-links in the TJ and enhances permeability of ions and small molecules, suggesting that both occludin and tricellulin are required for maintaining an intact epithelial barrier [30].

The canonical function of the claudins involves the regulation of paracellular transport of ions, small molecules and water. However, several claudins exhibit non-canonical functions. Mice lacking claudin-1 die within a day after birth and show defective formation of the epidermal barrier [31]. Targeted deletion of Claudin (CLDN)12 in mice reveals skeletal abnormalities, including an increase in articular cartilage and suppression of chondrocyte differentiation, indicating its role in bone homeostasis [32]. *Cldn*2 and *Cldn*12 form redundant and independent pores in colonic epithelium that facilitate paracellular calcium absorption. Double knockout of *Cldn*2/12 reduces calcium absorption and permeability in mice compared with single-null animals [33]. This double mutant is inflicted with hypocalcemia and decreased bone mineral density, which is absent in single knockout animals. Both *Cldn*2 and *Cldn*15 are indispensable for paracellular monovalent ion permeability, particularly sodium ions, in the intestinal mucosa of infant and adult mice [34]. The knockout of both *Cldn*2 and *Cldn*15 infant mice decreases nutrient uptake, leading to malabsorption and death [35]. *Cldn*18 deletion in mice gastric epithelium affects chloride flux but not TJ ion selectivity [36]. *Cldn*18-knockout mice develop intraepithelial neoplasia in the stomach. In particular, CLDN18 regulates gastric cell differentiation and signal transduction, including p53 and STAT [37].

The peripheral associated scaffolding proteins, zonula occludens (ZO-1, ZO-2 and ZO-3), are necessary for the assembly of TJ proteins. They connect TJ proteins with the actin cytoskeleton and signaling molecules [38]. The interaction between these proteins maintains TJ structure and function, yet the role of ZO proteins in the TJ is still unclear. ZO-1 deficiency does not affect initial formation of the TJ in mouse epithelial cells; however, lack of ZO-1 delays the recruitment of claudins/occludin and barrier formation in subsequent TJ formation [39]. The correct time course of TJ formation is critical for the timing of developmental processes, since ZO-1 knockout mice are embryonically lethal [40]. While ZO-2 deficiency does not affect TJ formation, ZO-2 knockout mice display severe phenotypes, including arrested cell growth and apoptotic cell death. ZO-3 deficiency shows normal TJ in cultured cells and mice [41].

## 2. Redox Regulation of the Permeability Barrier and Associated Disorders

Maintenance of redox homeostasis is essential for TJ proteins. The expression, localization and oligomerization of occludins are redox-dependent [42]. Sequence alignment shows that among the five conserved cysteines, two cysteines in the extracellular loop 2 (ECL2) form disulfide bonds under oxidative conditions. Such interactions are inhibited by the reducing agents, for instance dithiothreitol, or in hypoxic conditions in cultured kidney cells [43]. The intracellular GSH/GSSG ratio modulates the oligomerization of occludins. The occludin monomers and oligomers in equal numbers are present under physiological conditions, whereas the oligomeric assembly of occludin is disrupted by oxidative stress derived from hypoxia/reperfusion [44] or inflammation [45], leading to increased permeability of the barrier. Administration of H_2_O_2_ increases endothelial solute permeability and causes occludin rearrangement, including redistribution of the proteins on the cell surface and dissociation from ZO-1 [46]. The oxidized phospholipid, (Oxidized l-alpha-1-palmitoyl-2-arachidonoyl-sn-glycero-3-phosphorylcholine (OxPAPC)), reduces occludin mRNA/protein expression at cell–cell contact sites and increases occludin phosphorylation in vascular endothelial cells. OxPAPC also increases dextran flux and superoxide anion generation in bovine aortic endothelium. Scavenging of superoxide and H_2_O_2_ restores occludin gene expression by superoxide dismutase and catalase, respectively. Increased phosphorylation of serine and threonine in occludin induced by ROS modulates TJ structure and function [47]. Similar to occludin, claudin contains a disulfide bridge in its extracellular loop. Mutations of claudin-5 ECL2 increases FITC-dextran permeability, suggesting that ECL2 contributes to redistribution of claudin-5 and tightness of the paracellular space against ions and solutes in cultured kidney cells [48]. 

Oxidative stress-induced disruption of intestinal barrier function, such as paracellular permeability, is mediated by tyrosine kinase [49]. Tyrosine phosphorylation of junctional proteins is involved in the regulation of cell–cell adhesion and permeability [50,51,52]. Xanthine oxidase-induced oxidative stress causes a rapid increase in tyrosine phosphorylation of ZO-1, occludin, E-cadherin and beta-catenin in colon epithelial Caco-2 cell monolayers, which is accompanied by a decrease in trans-epithelial electrical resistance (TEER), which is indicative of disrupted barrier integrity [14]. The resulting dissociation of occludin-ZO-1 and E-cadherin and beta-catenin complexes from the cytoskeleton can be reversed by a tyrosine kinase inhibitor, genistein.

NO plays a dual role in the regulation of intestinal TJ function. At physiological intestinal NO concentrations, it interacts with cellular lipid and protein radicals formed during lipid peroxidation and protein oxidation [53,54]. At high concentrations of NO generated by iNOS, it can disrupt redox homeostasis by triggering signaling that leads to protein oxidation and lipid peroxidation. These higher concentrations of NO inhibit protein tyrosine phosphorylation through altering the intracellular GSH/GSSG ratio [4,9]. Post-translational modifications of signaling proteins, including nitration of tyrosine residues or S-nitrosylation of thiols, can be mediated by NO [55,56]. NO plays a role in the oxidative stress-induced alteration of intestinal barrier structure and function [4], such as in the treatment of Caco-2 cells with an NO donor (NOC5 or NOC12), by preventing the disruption of the tyrosine phosphorylation of junctional proteins and barrier function caused by H_2_O_2_ [9]. The up-regulation of iNOS and reduction in ZO-1, a target of microRNA-212, are caused by alcohol and an alcohol-containing diet in Caco-2 monolayer cells and mice, respectively. Knockdown of intestinal miR-212 inhibits hyper-permeability in the gut, indicating a close relationship between NO signaling and microRNA regulation in the development of the leaky gut [8]. Caco-2 cells treated with a mixture of pro-inflammatory cytokines cause epithelial barrier dysfunction, reduced expression and abnormal subcellular localization of TJ proteins, including ZO-1, ZO-3 and occludin. The fact that an NO scavenger, such as 2-(4-carboxyphenyl)-4,4,5,5-tetramethylimidazoline-1-oxyl-3-oxide (CPIO), mitigates these defects, is indicative of a direct effect of NO on pro-inflammatory cytokine-induced intestinal barrier impairment [13].

## 3. Role of Metabolism in Intestinal Homeostasis and the Pentose Phosphate Pathway (PPP) as a Therapeutic Target

Intestinal diseases are closely associated with altered metabolism. Metabolites, such as lipids, short chain fatty acids, and amino acids exert modulatory functions and beneficial effects in the intestine (Table 2). These modulators are considered promising druggable targets for chronic intestinal disorders. Pyruvate is an important intermediate metabolite of carbohydrate metabolism. Calcium pyruvate monohydrate, a stable pyruvate derivative, improves mucosal structure and reduces pro-inflammatory cytokines in a mouse model of colitis induced by trinitrobenzenesulfonic acid (TNBS) [57]. As key lipid components in the intestinal epithelium, sphingolipids play an important role in maintaining intestinal homeostasis, including maintaining barrier integrity, modulating nutrient uptake and regulating regeneration and differentiation of the intestinal mucosa [58]. Among the sphingolipids, ceramide and sphingosine-1-phosphate modulators have therapeutic efficacy in IBD [59,60]. Intestinal bacterial metabolites play a role in the development of intestinal disorders. Butyrate, a short-chain fatty acid, stimulates mature colonocytes and prevents the proliferation of Caco-2 cells by promoting differentiation of colorectal cancer cells [61]. It also stabilizes intestinal barrier function and reduces inflammation [62]. Reduction in butyrate-producing bacteria in the fecal microbiota and butyrate content in the gut are associated with IBD [63,64]. Reduced serum levels of tryptophan are found in IBD patients, indicating that tryptophan deficiency or degradation might contribute to the development of IBD [65]. Glutamine, an abundant amino acid, supports redox homeostasis by involving glutathione generation. Depletion of glutamine causes loss of villi, down-regulation of TJ proteins and enhanced barrier permeability [66]. It maintains intestinal barrier integrity through preventing methotrexate-induced barrier disruption in cells and animals [67,68].

Key metabolic genes play a role in intestinal diseases. Pyruvate kinase M2 (PKM2), which catalyzes the final step of glycolysis, is involved in cell survival and proliferation. Reduced levels of intestinal epithelial PKM2 are found in patients with Crohn’s disease and ulcerative colitis [82]. Several intestinal defects are found in intestinal epithelial PKM2-knockout mice, including increased intestinal inflammation, shortened colon, disruption of the TJ, enhanced inflammatory cytokine production and infiltration of immune cells [82]. Peptidoglycan recognition proteins (PGRP) regulate microbiota and intestinal homeostasis in fruit flies. The short lifespan of the PGRP-SD fly mutant is induced by overgrowth of *Lactobacillus plantarum* in its gut. Lactic acid derived from *L. plantarum* promotes ROS production through NOX, resulting in intestinal damage, enhanced proliferation of intestinal stem cells and dysplasia [83].

Induction of TP53-inducible glycolysis and the apoptosis regulator (TIGAR), a p53 target, is essential for cellular proliferation in the small intestine. TIGAR-knockout mice display reduced growth of regenerating intestinal crypts and transiently increased apoptotic intestinal cells after injuries to the intestinal epithelium induced by irradiation or genotoxic drugs [79]. These mutant mice have a slow recovery from ablation of the colon epithelium induced by ulcerative colitis. Elevated oxidative stress markers, such as lower levels of GSH and increased sensitivity to H_2_O_2_, are detected in the TIGAR-deficient baby mouse kidney epithelial cells. Increased lipid peroxidation is detected in the TIGAR-knockout mice. Supplementation with the antioxidant N-acetyl L-cysteine (NAC) or nucleosides fully restores the proliferation of the crypts and increases the GSH/GSSG ratio [79]. Inhibition of transketolase (TKT), an enzyme of the non-oxidative branch of the PPP by oxythiamine, impairs the crypts growth in TIGAR-deficient cells, which can be rescued by NAC [79].

IBD is a chronic inflammatory disorder, featuring a dysfunctional intestinal epithelial barrier. The major types of IBD are ulcerative colitis and Crohn’s disease. TKT is closely associated with IBD [84]. Intestinal epithelial cell-specific TKT-knockout mice exhibit growth retardation and spontaneous colitis. TKT deficiency causes mucosal erosion and elevated inflammatory cell penetration. Impaired TJ and barrier function, including increased permeability to FITC-dextran and reduced mRNA and protein expression of ZO-1 and occludin, are detected in the colon of TKT-knockout mice [84]. TKT abrogation increases intestinal epithelial apoptosis and modulates apoptotic genes, including up-regulation of BAX and down-regulation of Bcl-2, indicating that TKT maintains intestinal homeostasis by regulating energy production and programmed cell death.

As a human housekeeping gene and the rate-limiting enzyme of the PPP, glucose-6-phosphate dehydrogenase (G6PD) produces ribose-5-phosphate and NADPH for nucleic acid synthesis and reductive biosynthesis, respectively. G6PD, a hub for metabolic and redox reactions, is required for cell proliferation and organismal development [85]. Lack of G6PD activity due to gene mutations causes red cell-related clinical pathologies, including neonatal jaundice, favism, and drug or infection-induced hemolysis [86]. Severe G6PD deficiency disrupts lipid metabolism and the permeability barrier in nematode embryos leading to embryonic lethality [87,88].

G6PD-derived NADPH is important for the activity of NOX and NOS, since the generation of superoxide and nitric oxide from these enzymes are positively correlated with G6PD activity [89,90]. NOX1-deficient mice show intestinal pathology, including a defective mucus layer with bacterial infiltration into crypts and susceptibility to colitis, leading to mortality [19]. In addition to colon injuries, IBD is observed in NOX variants [20,91,92]. The characteristic and degree of colonic inflammation observed by histology and endoscopic examination in IBD is similar to chronic granulomatous disease (CGD), which is an immunodeficiency due to defective NOX activity [92]. Despite rare cases, G6PD deficiency mimics features of CGD, including impaired bactericidal ROS/RNS production and recurrent bacterial infections [93,94,95]. This suggests that G6PD may play a modulatory role in intestinal physiology. TIGAR suppresses glycolysis and reprograms glucose to the PPP through up-regulation of G6PD [96]. Since TIGAR is required for the intestinal epithelium through redox modulation [79], the assumption that the down-stream target G6PD may directly modulate intestinal physiology requires further elucidation.

## 4. Models of Intestinal Barrier Dysfunction and Associated Disorders

Advances in our understanding of the pathogenesis of intestinal barrier dysfunction has been provided by the investigation of different animal models. In these models, the intestinal barrier is mostly conserved across species in terms of structure and biology. Like in humans, the intestine barrier of rodents is sensitive to diet, microbiota and stresses [97]. Animal models of altered barrier function not only provide mechanistic details of intestinal leakage, but also allow for the assessment of potential therapeutics that target intestinal barrier dysfunction, through criteria such as enhancing barrier integrity and boosting immunity. Genetic knockout technologies [76,78,98], the introduction of a transgene [99,100] or exposure to chemicals [57] in animals, have been used to induce a particular intestinal pathology. Transfer of dysregulated T cells from mice with intestinal barrier dysfunction can trigger colitis in healthy animals [101].

Mice with IL-10 deficiency harbor intestinal microflora and trigger an enterocolitis in developing neonates. These mice display increased permeability in the ileum and colon without apparent histological defects. The impaired intestinal permeability is associated with increased secretion of interferon-gamma and tumor necrosis factor (TNF)-alpha in the mucosa. The intestinal inflammation caused by IL-10 deficiency is a response to normal enteric bacteria [76]. Axenic, luminal sterile IL-10-deficient adult mice do not develop enterocolitis, while they do, including elevated levels of IFN-gamma in cecal and colonic tissue, after inoculation with intestinal microflora. This suggests that IL-10 provides tolerance to bacterial antigens during bacterial exposure [77].

Junctional adhesion molecule A (JAM-A)-deficient mice display normal epithelium with enhanced leukocyte infiltration, lymphoid accumulation, increased permeability and reduced TEER [78]. Upon induction of colitis by DSS in JAM-A-deficient mice, increased epithelial cell growth is detected. In intestinal epithelial cells, JAM-A interacts with the tumor suppressor NF2 and LATS1 kinase, thereby initiating Hippo signaling and promoting cell proliferation [102], suggesting that JAM-A plays a role in intestinal homeostasis by modulating inflammation, barrier permeability and cell-cell contact in the intestine.

Cytokines trigger acute changes of the TJ in IBD that is mediated by cytoskeletal changes and chronic alteration of the TJ through modification of claudins. TNF-induced barrier damage is mainly caused by phosphorylation of myosin II regulatory light chains (MLC) by MLC kinase (MLCK). Knockout of MLCK in mice leads to TJ disruption, protein leakage, diarrhea and T cell activation [101]. Transgenic mice constitutively expressing MLCK in their intestinal epithelia induces MLC phosphorylation and enhances intestinal barrier permeability [99]. These mice exhibit normal growth, intestinal histology and TJ organization, yet they have an induced mucosal immune response, including recruitment of CD4(+) lymphocytes in the lamina propria and increased expression of TNF and IFN-gamma. Although these mice do not develop disease under pathogen-free conditions, they experience reduced survival and an accelerated and severe form of colitis upon challenge with colitis-inducing dysregulated immune cells, suggesting that impaired function of the intestinal barrier is not sufficient to cause intestinal diseases.

Intestinal barrier dysfunction can alter the tolerance of exogenous antigens and xenobiotics, causing chronic inflammation. The multi-drug resistance protein (MDR), a xenobiotic transporter, is involved in the maintenance of intestinal homeostasis. Mdr-1-knockout mice develop colitis under pathogen-free conditions. Increased ion transport, decreased TEER in the colon, reduced phosphorylation of TJ proteins and increased expression of cyclooxygenase-2 and iNOS, are observed in these mice [98]. The increased levels of these inflammatory proteins and translocation of bacteria across the intestine are associated with disease severity [98], indicating that altered function in the intestinal barrier without immune dysfunction in mdr-1-deficient mice is sufficient for contributing to colitis.

The transcription factor nuclear factor (NF-κB) is a key regulator of pro-inflammatory and immune responses in the intestinal epithelium. Enteric pathogenic bacteria and cytokines in intestinal epithelial cells induces NF-κB activation. NF-κB-derived pro-inflammatory cytokines, such as IFN-gamma and TNF-alpha, impair barrier function by increasing TJ permeability and down-regulating occludin and ZO-1 [103,104]. T-cell induced internalization of occludin and claudin-1 is mediated by NF-κB [100]. Transgenic mice expressing epithelial-specific IκBα, a super suppressor of NF-κB, exhibit reduced T cell-induced diarrhea, TEER and trans-mucosal flux of dextran and bovine serum albumin, indicating that NF-κB inactivation can ameliorate defective barrier function in the intestinal epithelium, including loss of serum proteins.

Rodent intestine models are of great importance in elucidating the mechanism of intestinal barrier diseases [97]. However, due to regulations regarding animal welfare and ethical considerations, increasingly, non-mammalian models have been developed and used as alternatives models for assessing intestinal barrier function. One of the simple invertebrate model organisms is *Caenorhabditis elegans*. It is a free-living soil nematode and commonly used owing to advantages in the laboratory, including convenience of culture, transparent body for microscopy, short generation time, invariant cell lineage and tractable genetics [105]. The simple epithelia in the pharynx and intestine during the embryonic and larval stages provides essential information regarding the development and maintenance of intestinal epithelial cell junctions. Forward genetic screening and reverse genetic approaches in *C. elegans*, such as RNAi, facilitate studying the role and function of TJ proteins. *C. elegans* possess several claudin-like proteins [106]. Claudin-like in Caenorhabditis (CLC-1) is expressed in the epithelium in the pharyngeal section of the intestine. Knockdown of *clc-1* disrupts barrier function, such as increased diffusion of dextran to the outside of the lumen of the digestive tract, while CLC-2 is involved in hypodermis barrier function [107]. The intestine of *C. elegans* can undergo an epithelial response to luminal stressors. Unlike the immune complexity in mammals, no specialized leukocytes, such as lymphocytes and macrophages, are found in the *C. elegans* gut [108]. Immunity in *C. elegans* relies on the gut epithelial barrier, which is important in regulating its lifespan. Hence, rather than extensive pre-clinical testing, it has become an attractive model for assessing therapeutic interventions, such as probiotics, against gut barrier infections [108].

## 5. Conclusions

Impaired TJ structure and gut barrier dysfunction are the culprits of intestinal disorders. The disruption of the barrier by RS and toxic agents causes hyper-permeability and an inflammatory response. In contrast, the protection of intestinal barrier integrity mediated by redox homeostasis, metabolism and nutritional factors is beneficial to general gut health. The underlying mechanism of intestinal homeostasis requires further investigation by appropriate animal models for addressing the causal effects of the TJ in a number of disorders. The mechanistic details regarding the regulation of the TJ will provide a wealth of information for developing new therapeutic and diagnostic approaches.

## Figures and Tables

**Table 1:** Examples of factors that disrupt the intestinal barrier.

Agents	Model	Findings	Reference
Alcohol	Caco-2 cells mice	Induces intestinal miR-212 expression, iNOS, ZO-1 down-regulation and intestinal hyperpermeability Knockdown of miR-212 prevents intestinal hyperpermeability	[8]
Hydrogen peroxide	Caco-2 and MDCK monolayer cells	H_2_O_2_-induced TJ disruption is sensitized by tyrosine phosphorylation mutation in occludin Increases inulin permeability, redistributes occludin and ZO-1 Decreases in TEER, increases paracellular permeability to dextran Disrupts the intercellular junctional localization of ZO-1 and induces tyrosine phosphorylation of ZO-1	[9,10]
particulate matter	Caco-2 cells	Increases oxidative stress (4HNE adducts) Decreases levels of ZO-1, claudin-1, and desmocollin	[11]
particulate matter	Caco-2 cells C57BL/6 mice	Induces cell death, mitochondrial ROS, oxidant-dependent NF-κB activation, permeability, disruption of tight junctions in Caco-2 cells Increases intestinal permeability and IL-6 mRNA, reduces ZO-1 mRNA/protein in the small bowel in mice	[12]
Pro-inflammatory cytokines	Caco-2 cells	Induces epithelial barrier dysfunction, reduces expression and abnormal subcellular localization of ZO-1, ZO-3 and occludin	[13]
Xanthine oxidase and xanthine	Caco-2 cells	Rapid increase in tyrosine phosphorylation of ZO-1, occludin leading to dissociation of TJ *C-src* inactivation delays ROS-induced TJ disassembly and function Decreases TEER Increases inulin permeability Redistribution of occludin and ZO-1	[14,15]

**Table 2:** Examples of factors that protect the intestinal barrier.

Agents	Model	Findings	Reference
Butyrate	Colonocytes	Exerts anti-inflammatory effect through inhibiting histone deacetylase Provides immune protection through stimulating G-protein coupled receptors	[62,63,64]
Cyba (p22^phox^)	Mouse	*Cyba* mutation increases susceptibility to DDD-induced colitis: shorter colon length, increased cell infiltration in the mucosa, and loss of crypts *Cyba* variant has a thinner mucus layer in the colon and an increase in penetration of bacteria in the crypts Reduces ROS production causes gut flora dysbiosis in *Cyba* mutant	[19]
Glucagon-like peptide-2 (GLP-2)	Caco-2 cells	Increases transepithelial electrical resistance Increases occludin and ZO-1 GLP-2 diminishes TNF-a-induced TJ function, expression and localization	[69]
Glutamine, Arginine	Caco-2 cells	Prevents methotrexate (MTX)-induced barrier disruption by restoring TEER, FITC-dextran permeability and increasing ZO-1 and occludin expression	[67]
Glutamine	Sprague Dawley rats	Prevents MTX-induced gut barrier disruption by enhancing occludin/claudin-1/ZO-1 expression and reducing FITC-dextran permeability	[68]
Hypoxia Inducing Factor (HIF)	T84, 293T and colon epithelial cells	Reduces claudin-1 in HIF1β-deficient cells PHD3 stabilizes occludins PHD3 is inversely correlated with ulcerative colitis	[70,71]
HIF hydroxylase inhibitors or HIF-1α stabilizer	murine models	Ameliorates IBD in murine models: reduces inflammatory lesions and pro-inflammatory cytokines Reduces apoptosis in intestinal epithelium and improves barrier function	[72,73,74,75]
IL-10 gene	Mouse	IL-10 deficiency increases permeability in ileum and colon IL-10 deficiency increases secretion of interferon-gamma and tumor necrosis factor-alpha in the mucosa upon bacteria exposure	[76,77]
JAM-A	Mouse	JAM-A deficiency enhances leukocyte infiltration, lymphoid accumulation, increases permeability and reduces TEER	[78]
NO donors (NOC5, NOC12)	Caco-2 cells	Restores the disrupted barrier function by enhancing TEER and reduces paracellular permeability) Prevents H_2_O_2_-induced tyrosine phosphorylation of ZO-1	[9]
NAC	Mouse intestinal crypt culture	Restores proliferation of the intestinal crypts Increases GSH/GSSG ratio in cell culture	[79]
Pyruvate	Mice	Recovers mucosal cytoarchitecture Reduces pro-inflammatory cytokines (IL-1, IL-6, IL-17, IL-23) and iNOS in TNBS-induced colitis model	[57]
Qucertin	Caco-2 cells	Increases TEER Reduces paracellular permeability Promotes assembly of occludin, claudin and ZO-2	[80]
Quercetin nanoparticles	Wistar rats	Reduces the disease severity in dextran sulfate sodium (DSS)-induced colitis Enhances antioxidant status, occludin, MUC-2 and JAM mRNA expression Decreases iNOS, COX2, and proinflammatory cytokines	[81]
Sphingolipids (Ceramide, sphigosine-1-phosphate)	Clinical data	Maintains Intestinal barrier integrity, nutrient absorption Regulates regeneration and differentiation of the intestinal mucosa	[58,59,60]
Tryptophan	Clinical data	Deficiency is correlated with IBD	[65]
